# Comparing teacher and student perspectives on the interplay of cognitive and motivational-affective student characteristics

**DOI:** 10.1371/journal.pone.0200609

**Published:** 2018-08-15

**Authors:** Sina A. Huber, Tina Seidel

**Affiliations:** Technical University Munich (TUM), School of Education, Munich, Germany; Northwestern University, UNITED STATES

## Abstract

For students, cognitive and motivational-affective characteristics are the most powerful prerequisites for successful learning. For teachers, judgments on their students’ characteristics shape how they plan and implement instructional activities in order to offer individual learning support. On the student side, research is starting to find out more about the interplay of different characteristics within individual students. On the teacher side, studies still regard teacher judgment accuracy of only single characteristics. By taking a person-centered approach, regarding N_S_ = 503 students and their N_T_ = 41 mathematics and languages arts teachers, our manuscript joined teacher and student perspectives on student characteristics interplay and suggests methodology to compare them. We found that student assessments suggested ample diversity regarding this interplay–and teachers did not perceive this. In their views, “homogeneous” sets of average characteristics were dominant. Findings suggest addressing students’ views and the diagnosis of their characteristics in teacher education to enable individual support.

## Introduction

Individual student characteristics are crucial for student learning. Cognitive and motivational-affective student characteristics such as general cognitive ability or self-concept have powerful effects on their learning outcomes [1,2]. Their role in student learning is especially multifarious since these different characteristics are interrelated. Studies have found, for instance, that self-concept, interest and achievement have mutually reinforcing effects over time [3]. However, these complex interactions of different cognitive and motivational-affective characteristics are not fully understood yet. Furthermore, it remains unclear what existing effects among characteristics mean for individual students, e.g. [4]. Hence, recent research has explored the interaction of different student characteristics from a person-centered point of view. This line of research has identified large groups of students with incoherences in the interplay of characteristics: Studies find students that are able but not confident [5], knowledgable but not interested [6], or self-efficacious but only moderate achievers [7]. Since these studies still focused on grouping students based on their similarities instead of exploring the full extent of their differences, from a student side, it remains unclear how diverse the within-student interaction of student characteristics really is. Our study complements this line of research by adding a new perspective of within-student characteristics diversity to the understanding of the complex interaction of characteristics while suggesting methodology to do so.

For teachers, knowledge of student characteristics is essential because of the characteristics’ central role in student learning [8]. Through adaptive teaching, teachers are expected to support their students according to their individual learning preconditions [9]. Yet, teachers can only plan and implement individualized teaching activities if they are aware of their individual sets of characteristics. This diagnostic process has long been described as a main challenge in teaching [10] and is not yet fully understood. We know that teachers vary in their ability to accurately judge single student characteristics [11]–with great variation depending on which characteristic is being judged [12]. Furthermore, we are beginning to understand some of the biases that influence this judgment process [13]. These biases refer to teacher beliefs and stereotyping, but also include regression or sampling effects in their judgment of single student characteristics. Yet, it remains uncertain how teachers perceive the interplay of different cognitive and motivational-affective characteristics in their individual students. From a teacher perspective, our manuscript offers a new insight on teacher judgments of student characteristics by measuring their view on the diversity of characteristic interaction and juxtaposing it with their students’ assessments.

Hence, our study explores the diversity in student characteristics from two new angles. First, we employ a new set of person-centered methods to examine student characteristics diversity in more detail. Second, we juxtapose our findings based on student assessment with their teachers’ perception of diversity to shed light onto the teachers’ perspective on characteristics diversity.

### Individual student characteristics

In the 21st century, individual differences and diversity of learners are key elements in our discussion about learning and teaching [14]. Diversity with respect to students’ background such as socio-economic or migration background or gender are connected to differences in students’ characteristics like self-concept or prior achievement, e.g. [15]. Research suggests, it is through those student characteristics that learning is influenced by students’ background diversity. Some scientists, for instance, found that treating self-perceptions has a mediating effect on minority students’ achievement gap [16].

The importance of different cognitive and motivational-affective student characteristics for learning and achievement has been extensively studied [17]. A number of student characteristics have been found to have an essential impact on student learning. First, general cognitive ability is cited as the most predictive factor in students’ success [1]. Beyond that, prior achievement in an academic subject area has been found to be closely linked to students’ future performance [18]. Research increasingly highlights that beyond cognitive features, motivational-affective characteristics are crucial for students’ learning as well [19]. Subject-related interest, defined as a predisposition toward enjoying and valuing a particular academic subject domain [20], predicts academic achievement [21]. Additionally, students’ subject-related self-concept of ability, defined as an individual’s self-perception regarding abilities concerning this academic subject matter and formed through experiences with the environment [22], is found to be essential for learning success [23,2].

### The interplay of student characteristics: Incoherences and measurement

#### Incoherences in the interplay of characteristics

Looking beyond the effect of single characteristics on student learning, studies have identified how different sets of characteristics relate to educational processes and outcomes. Researchers found that certain combinations of interest, self-efficacy, and prior knowledge were related to higher learning outcomes [7]. Furthermore, another study could show that the combination of cognitive and motivational-affective characteristics was critical for students’ perception of their learning environment [6]. Scientists also recognized that certain configurations of characteristics were connected to student achievement and engagement [5]. The specific composition of characteristics also made a difference regarding students’ involvement in class and their situational learning motivation [24,25].

When investigating sets of characteristics, these studies found that, for many students, the different characteristics did not align, revealing what we will call ‘incoherences’ in the interaction of student characteristics. One study categorized 30–45% of students into inconsistent patterns [5]. For almost all of their types, there was only partial alignment. In another study, over half of students belonged to fragmented profiles [6]. Some studies have even started explicitly to highlight pseudo-concurrences in characteristics interplay [4]. Yet, to our knowledge, no study has focused on how different students really are with respect to their within-sets of student characteristics. This ‘student characteristics diversity’ will be addressed in our study.

#### Measuring diversity and individual differences in the interplay of characteristics

Uncovering these incoherences in the interaction of student characteristics is a question of methodology as well. The interplay of student characteristics has largely been explored by studying how the characteristics as variables were interrelated. Whether studies use regression or more complex modeling including latent constructs, e.g. [3], many of the variable-level connections found in these studies are low to moderate. In one study, for instance, researchers found only small effects of cognitive and motivational-affective characteristics on learning outcome when taking a variable-centered approach, but uncovered the strong relationship of student characteristics to learning gains employing person-centered analyses [7]. Person-centered methods do not analyze aggregated variable values of a population, but persons or objects with their set of variables values. Instead of assuming a homogeneous population, person-centered methods allow for identifying distinct subgroups [26]. In this property, they seem ideal for the within-student study of diversity and individual differences.

Configural frequency analysis offers a way for contingency table data to identify over- and under-frequented patterns by comparing observed to expected cell count [27]. Since its goal is not the grouping of all individuals, it allows exploring the diversity in a population more so than other person-centered methods. It has been used in psychological research [28], but has not been connected so far with student diversity analysis.

Diversity measurement, in general, is also known to other disciplines. Biology, for instance, uses diversity indices to measure and compare the amount of diversity in naturally occurring systems, like forests [29]. A quite commonly used index in biology is the Shannon-Wiener Diversity Index which allows to measure diversity in nature similarly to information contained in a message [30]. Measuring diversity with a diversity index has also spilled over into other disciplines, for instance economics [31]. In our study, we want to transfer this knowledge from biology to the field of educational psychology and connect variable- and person-centered approaches. We expect through this innovative methodology to uncover–besides existing knowledge of (low to moderate) positive connections–considerable diversity in within-student characteristics interplay.

### Teacher perceptions of within-student characteristics and their diversity

Teacher perceptions of their students’ characteristics greatly affect student learning [32]. They are essential prerequisites to the long-standing goal of adaptive teaching for individual student support [33]. However, research is just starting to understand teachers’ perspective on their students’ individual differences. Findings on judgment accuracy and the process of teacher judgments hint that teachers’ views of their students’ characteristics might differ from what student assessments of these characteristics reveal. This is why our study juxtaposes teacher with student perspectives on the interplay of student characteristics.

#### Individualized instruction

Teaching must be tailored to different students with different characteristics [34]. This need to adapt instruction has been studied for more than a century [33,35]. Yet, with the rise of diversity as an issue in the educational debate, recent policies, e.g. [36], increasingly call for using assessments to “individualize instruction for students with diverse learning needs” [9]. Indeed, teachers plan and implement instructional activities according to their appraisal of their students’ dispositions [37]. They choose tasks and assemble learning groups according to their judgment of students’ characteristics [38]. The central role of teachers’ ability to diagnose each individual student’s unique set of learning prerequisites is therefore indisputable. Yet, research examining if teachers are indeed able to do so is still not satisfactory. According to current research, two lines of research exist: First, research regarding teacher judgment accuracy and its conditions and second, research regarding the process of those judgments [10]. However, both lines cannot yet tell if teachers see the within-student diversity of characteristics.

#### Teacher judgment accuracy

Large meta-analyses report that teachers are generally able to accurately assess their students’ achievement (overall agreement of teacher perceptions and student assessment r = .65 [39,11]). However, studies point out that even in these meta-analyses, large parts of variance remain unexplained [40]. Also, research suggests teachers to have more difficulty accurately judging their students regarding other student characteristics such as intelligence or motivation [12]. Hence, research on teacher judgment accuracy yields mixed findings for different characteristics.

Additionally, the measurement of judgment accuracy itself remains an issue. Findings juxtaposing the different methods uncover discrepancies due to measurement and conclude that it is difficult to speak of general judgment accuracy [12]. In these mixed findings on teacher judgment accuracy for different characteristics, we cannot make clear predictions on the judgment of characteristics interaction based on this line of research alone.

#### The complex process of teacher judgments

Studies find that teacher judgments of different characteristics are tied closer than the actual characteristics. For instance, teachers expect students who they rate low on prior achievement to also be less motivated or less engaged [40,41]. This agrees with the finding that human judgment has the tendency to overgeneralize yielding the ‘halo effect’ [13]. This effect occurs when the judgment of a single characteristic overshines the perception of others. It is known from different areas of psychology [42].

Research has further started to uncover the complexity of judgment processes. It identified different sources of bias in teacher judgments including teacher beliefs, stereotyping, regression and sampling effects [13]. In general, teachers do not seem to believe student assessment is closely connected to student diversity [9]. Also, knowing about relationships on group-level, teachers might infer that characteristics align for individuals–a misconception called the pseudo-contingency illusion [4]. Additionally, stereotyping connects teacher beliefs to student background variables such as gender, ethnic or socio-economic background, e.g. [43]. Finally, it also remains somewhat unclear how classroom activities shape teacher perceptions. Studies have found that not necessarily the label (e.g. ADHD) but students’ actual behavior influences teacher impressions and predictions [44]. Research examining what teachers actually notice in classrooms is only at its beginning and studies hint that this might also vary between teachers [45]. Because of these many influences on the teacher perceptions, we still do not know if teachers really see the individual students to adequately individualize their teaching to it.

This is why our study examined the interplay of student characteristics from a teacher’s perspective as well. Via our person-centered methodology and its property of allowing statistical comparisons between student and teacher perspective, we expect to describe patterns of teachers’ views on the within-differences in their students–as a prerequisite for offering individual learning support.

### Characteristics diversity in different academic subject areas

On the students’ side, findings regarding student characteristics and their interaction often vary between academic subject areas. For instance, interest is tied more closely to achievement for mathematics than for language arts [21]. Also, self-concept and grades are connected closer for mathematics than for language arts [23]. Furthermore, general cognitive ability explained more of the variance in mathematics achievement than in any language instruction [1]. Regarding the teachers’ perspective, on the other hand, their perceptions are often studied regarding a specific academic subject area, e.g. [46], or across academic subjects on a primary school level, e.g. [37], but seldom systematically compared between different academic subject areas. Our study examined the diversity in student characteristics interplay with regard to two distinct prominent academic subject areas of secondary education, mathematics and language arts to find if student and teacher perspectives are subject-specific.

### Research questions

Our study investigated the diversity in the within-interplay of the four characteristics: general cognitive ability, prior achievement, interest, and self-concept. In a methodological triad, three aspects of this diversity are considered:

- Correlation-like pairwise connections between variables were explored from a student and teacher perspective to verify that variable-centered perspectives were in line with existing research.
- The Shannon-Wiener Diversity indices adapted from diversity measurement in theoretical biology quantified the amount of diversity in the interaction of student characteristics from student and teacher perspective. Related t tests allowed for statistical tests between both perspectives and between academic subject areas.
- Configural frequency analyses further examined the distribution of types of students with regard to their sets of characteristics from a student and a teacher point of view.

For our following three research questions, hypotheses regarding all three methodological approaches (a-c) above were considered:

RQ I. How much within-student diversity is found in the interplay of the four student characteristics?

Conjectures: a) Following variable-centered research on the relationship of the different student characteristics, we suspected to find low to moderate positive connections between the different characteristics in our variable-centered approach. b) Considering those low connections on variable level, further person-centered analyses were conjectured to yield high within-student diversity and c) few over-frequented types (meaning sets of characteristics that appeared more often than statistically expected).

RQ II. How much within-student diversity do teachers perceive?

Conjectures: Regarding research on the possible biases in teacher judgment processes and in line with prior findings, a) we expected teacher perceptions of the four characteristics to be tied more closely. b) Diversity index comparisons were therefore expected to show significantly less diversity in teacher perceptions compared to student assessment and c) possibly over-frequented homogeneous student types (meaning within-student characteristics are homogeneously judged as low/medium/high).

RQ III. Can within-student characteristics diversity be found differently in two academic subject areas?

Conjectures: In alignment with previous findings regarding the comparison of both academic subject areas, for the student perspective, a) we expected a closer relationship of student characteristics in mathematics than in language arts yielding b) higher measured diversity and c) fewer or less homogeneous types for language arts. Regarding teacher perceptions, we did not make conjectures for academic subject area differences.

## Method

The research of this manuscript was approved by Bayerisches Staatsministerium für Bildung und Kultus, Wissenschaft und Kunst (Bavarian Ministry of Education) regarding content, participant and data protection (§24 BaySchO). Especially, all relevant articles of national and state-level data protection laws (BDSG, BayDSG) have been adhered to. All participants of this study (teachers as well as students and their legal guardians) have given their written informed consent (Art 15 Abs 2,3 BayDSG).

### Sample

This study examined *N*_*S*_ = 503 eight-grade students from *N*_*C*_ = 20 classrooms of a high teaching track (German Gymnasium) and their *N*_*T*_ = 41 mathematics and German language arts teachers (one language arts class was taught by two teachers.). All but three classrooms were coeducational with 51.2% female students. Students’ mean age was 13.41 years (*SD* = 0.61). All classrooms were located in middle class urban and suburban areas of southern Germany (Mother’s mean ISEI 55.75 (*SD* = 19.63), father’s mean ISEI 60.48 (*SD* = 20.74), the International Socioeconomic Index (ISEI) scores of occupational status are derived from International Standard Classification of Occupations (ISCO) scores developed by the United Nations International Labour Office [47] and range from a minimum of 16 to a maximum of 90 [48]). The student families had, on average, a high educational background (76.4% of mothers completed at least upper secondary education, 60.0% attained tertiary education; 95.2% of fathers completed at least upper secondary education, 68.3% had tertiary educational degrees). The majority (67.3%) of students was born in Germany with no migration background (29.1% of students were second-generation immigrants, 3.6% were first-generation immigrants). Most students lived with two parents at home (86.6%). The two groups of teacher participants, *n*_*TM*_ = 20 mathematics and *n*_*TL*_ = 21 German language arts teachers, have mean ages of 40.24 (*SD* = 10.91) and 40.70 years (*SD* = 10.10), and averages of teaching experience of 11.26 (*SD* = 10.40) and 11.42 years (*SD* = 8.15), respectively.

### Design

Data for the study was collected three months into the academic year of 2013/14 over a period of two weeks. Within this period, teachers and students were asked to answer questionnaires of the research team. Mathematics and language arts teachers also independently filled out teacher questionnaires on their perception of their students’ characteristics and sent back their documents in separate, sealed envelopes. At this point of measurement, teachers and students had had three months of teaching and learning together on which their assessments are based. Teachers administered the student questionnaires and were instructed to follow a strict routine that included a fixed testing time and privacy of students’ answers. After assessment, one student of the classroom collected the student questionnaires in a sealed envelope that could not be checked by their teachers.

### Instruments

The general cognitive ability of students was tested using a subscale of the Kognitiver Fähigkeitstest (KFT, cognitive ability test), a test frequently used in Germany [49]. The subscale comprised 25 figure analogy items (α = .89). This subscale was chosen instead of verbal and numerical subscales of the KFT to minimize academic subject area bias for this measure. Teachers provided the students’ mathematics and German language arts grade from the previous school year as a measure of their prior achievement. In Germany, grades range from 1 (best grade) to 6 (worst grade). For this study, the grades were re-coded ranging from 0 to 5 with low values indicating low prior achievement and high values high prior achievement. Students were administered four items on their interest for mathematics (α = .88) and three items on their interest for language arts (α = .82). Items were taken from the 2012 and 2009 questionnaires of the Program for International Student Assessment (PISA). An example item was: "I do mathematics because I enjoy it." (Range: 1 = strongly disagree to 4 = strongly agree [50]). The students’ self-concept regarding mathematics and language arts was collected using PISA scales of six items (α = .92) and five items (α = .82), respectively, from the 2012 and 2009 PISA questionnaires, for example "I learn things quickly in German class" (Range: 1 = strongly disagree to 4 = strongly agree [51,52]). Teachers were asked to rate each individual student’s general cognitive ability, achievement, interest and self-concept regarding mathematics on a scale from 1 “low” to 3 “high” in the teacher questionnaire.

### Data analysis

Student characteristics data was recoded for its distribution to match that of teacher data to eliminate scale level effects in the analyses most closely. For each characteristic, the top and bottom fourth of students were considered "high" and "low" in this characteristic, the remaining students received "medium" in this characteristic and students were assigned the values from 1 “low” to 3 “high” accordingly. This resulted in the symmetrical distribution that made data most similar to the distribution of teacher perceptions (see Results).

#### Interacting characteristics: A variable-centered approach

To account for the multi-level structure of the data (students within classrooms) and for easier interpretability and coherence, multi-level linear regression analyses were performed with each pair of student characteristic variables [53] for teacher and student data in their integer form introduced above. Appropriate simple, multi-level random intercept, or multi-level random slope regression model were chosen based on model fit. Since standardized scores were regarded, we reported β values in a correlation-like table leaving full results in S1 Appendix, which also discusses appropriateness and interpretation of analyses and results.

#### Measuring characteristics diversity: A person-centered approach

For each student, student assessment as well as teacher perception on the four student characteristics was displayed as a four-dimensional vector, their diversity pattern, with a total of 81 possible patterns.

The Shannon-Wiener diversity index *H* is a way to summarize observed frequencies that increases when there are more different patterns and when they appear more equally distributed. It was computed as:

$$H=-\sum_{k=1}^{K}p_{k}\ln\;\left(p_{k}\right),$$

where *p*_*k*_ is the observed proportion of individuals with diversity pattern *k* and *K* is the number of total patterns present according to student assessment and teacher perceptions in either academic subject area [54]. Moreover, the measure of evenness, *J* = *H*/*H*_*max*_, relating *H* to its theoretical maximum *H*_*max*_ = *ln*(*K*), and connected measures were calculated to investigate these diversity patterns. Diversity indices were statistically compared between teachers and student and between mathematics and language arts using Hutcheson’s student's t tests (cf. S1 Appendix).

Finally, we compared the observed occurrences of patterns to the expected amounts via configural frequency analysis. Student characteristics patterns that appeared significantly more often than expected, so-called types, were identified for both, the student and the teacher perspective in both academic subject areas. Types in configural frequency analysis can be identified employing local χ2 tests relating observed frequencies (n_o_) to expected frequencies (n_e_) [55]. More detail can be found in S1 Appendix.

## Results

Exploring the two perspectives on the within-student diversity in characteristics, we found considerable differences between student and teacher perspectives in both academic subject areas, as well as small but noticeable differences between subject areas. For reference, descriptive statistics regarding student and teacher instruments are reported in Table 1.

**Table 1:** Descriptive results of student and teacher questionnaires.

**Student questionnaires**	M	SD	Min	Max
General cognitive ability (GCA)	17.81	5.24	0.00	25.00
Prior achievement mathematics (ACH)	2.87	0.99	0.00	5.00
Interest for mathematics (INT)	2.20	0.78	1.00	4.00
Self-concept regarding mathematics (SC)	2.47	0.84	1.00	4.00
Prior achievement language arts (ACH)	3.00	0.77	0.00	5.00
Interest for language arts (INT)	2.84	0.91	1.00	4.00
Self-concept regarding language arts (SC)	2.85	0.52	1.00	4.00
**Teacher questionnaires**		low	medium	high
*Mathematics teachers*				
General cognitive ability (GCA)		16.8%	56.3%	26.9%
Prior achievement mathematics (ACH)		27.5%	51.0%	21.5%
Interest for mathematics (INT)		20.9%	51.4%	27.7%
Self-concept regarding mathematics (SC)		20.3%	58.0%	21.7%
*Language arts teachers*				
General cognitive ability (GCA)		16.6%	51.4%	32.0%
Prior achievement language arts (ACH)		22.2%	55.2%	22.6%
Interest for language arts (INT)		23.7%	45.4%	30.9%
Self-concept regarding language arts (SC)		16.6%	59.9%	23.5%

### Interacting student characteristics: Variable-centered results

An overview of standardized regression coefficients $\beta_{ij}^{DS}$ from pairwise regressions can be found in Table 2. In the text, regression coefficients are indexed by data D (student assessment S, teacher perception T), academic subject area S (mathematics M, language arts L), and characteristics (i,j) (G: general cognitive ability, A: achievement, I: interest, S: self-concept). Model comparisons resulted in a predominance of multi-level random intercept regression models. Model fit (Table A in S1 Appendix) and full modeling results including tests of significance (Table B in S1 Appendix) can be found in S1 Appendix. Based on [56] and [57], we are labeling coefficients with absolute values between 0.3 and 0.6 “moderate” and those lower and higher “weak” and “strong” accordingly for the purpose of discussion.

**Table 2:** Relationship between cognitive and motivational-affective student characteristics regarding mathematics and language arts according to student assessment and teacher perception.

	Mathematics				Language Arts			
	GCA	ACH	INT	SC	GCA	ACH	INT	SC
*Student Assessment*								
GCA	-	0.23***	0.21***	0.24***	-	0.18***	0.13**	0.12**
ACH	0.23***	-	0.34***	0.52***	0.18***	-	0.26***	0.32***
INT	0.21***	0.33***	-	0.54***	0.15*	0.26***	-	0.19***
SC	0.23***	0.50***	0.55***	-	0.12**	0.32***	0.18**	-
*Teacher Perception*								
GCA	-	0.71***	0.56***	0.51***	-	0.62***	0.43***	0.43***
ACH	0.68***	-	0.56***	0.54***	0.62***	-	0.57***	0.50***
INT	0.57***	0.58***	-	0.41***	0.42***	0.55***	-	0.46***
SC	0.48***	0.53***	0.39***	-	0.40***	0.48***	0.44***	-

Table shows standardized regression coefficients β from pairwise multi-level random slope regressions. Cognitive domain: GCA: general cognitive ability, ACH: achievement; motivational-affective domain: INT: interest, SC: self-concept

* p < .05

** p < .01

*** p < .001 (To account for multiple comparisons, only results at p < .001 and smaller are regarded to be significant.)

On the students’ side, results showed predominantly weak connections between the four student characteristics for both academic subject areas ranging from $\beta_{GS}^{SL}=\beta_{SG}^{SL}=0.12$ (which even did not vary significantly from zero, accounting for an adjusted significance level due to multiple comparisons) to $\beta_{AI}^{SM}=0.34$ with only the connection of self-concept with achievement and interest in mathematics at a moderate level ($0.50\leq \beta_{ij}^{SM}\leq 0.55$). In mathematics, the relationship between general cognitive ability and achievement, i.e. the connection within the cognitive domain, was weak at $\beta_{GA}^{SM}=\beta_{AG}^{SM}=0.23$. As opposed to this, relationships within the motivational-affective domain were the strongest over all pairs of characteristics in student assessment ($\beta_{IS}^{SM}=0.54,\beta_{SI}^{SM}=0.55$). For mathematics, connections between cognitive and motivational-affective domains ranged from a weak $\beta_{GI}^{SM}=0.21$ for general cognitive ability and interest to a moderate $\beta_{AS}^{SM}=0.52$ for achievement and self-concept. For language arts, all pairwise connections were even weaker for student assessments, with several ones not significantly differing from zero (see Table 2). Overall, the connection within domains was weak for both, the cognitive and the motivational-affective domain ($\beta_{GA}^{SL}=0.18,\beta_{AG}^{SL}=0.18,\beta_{IS}^{SL}=0.19,\beta_{SI}^{SL}=0.18$). Additionally, across-domain relationships were also rather weak ranging from $\beta_{GS}^{SL}=0.12$ (n. sign.) for general cognitive ability and self-concept to $\beta_{AS}^{SL}=0.32$ for achievement and self-concept.

From the teachers’ perspectives, on the other hand, results revealed moderate to strong connections between student characteristics ranging between $\beta_{SI}^{TM}=0.39$ for self-concept and interest and $\beta_{GA}^{TM}=0.71$ for general cognitive ability and achievement, both in mathematics. For both academic subject areas, connections within the cognitive domain were strongest ($\beta_{GA}^{TM}=0.71,\beta_{AG}^{TM}=0.68{,\beta }_{GA}^{TL}=\beta_{AG}^{TL}=0.62$). In mathematics, connections within the motivational-affective domain the weakest but still moderate ($\beta_{IS}^{TM}=0.41,\beta_{SI}^{TM}=0.39$). Regarding language arts, these were slightly stronger and at the same level as cross-domain connections ($0.40\leq \beta_{ij}^{TL}\leq 0.57$).

### Measuring diversity in the interplay of student characteristics: Person-centered results

#### Measuring diversity: The Shannon-Wiener diversity index

The students’ perspective showed that 59 different diversity patterns (73%) in mathematics and 74 (91%) in language arts of the *K*_*max*_ = 81 possible diversity patterns could be observed. Diversity index measures are given in Table 3. The Shannon-Wiener diversity index for student assessment was *H* = 3.71 with an evenness of *J* = 0.91 for mathematics and *H* = 3.98 (*J* = 0.92) for language arts. Teachers, on the other hand, perceived 44 (52%) of the 81 diversity patterns in mathematics and 52 (64%) in language arts. The Shannon-Wiener diversity index was *H* = 3.17 (*J* = 0.84) for mathematics teachers and *H* = 3.33 (*J* = 0.84) for language arts teacher perceptions. When comparing student and teacher perspectives, t test results indicated that teachers perceived significantly less diversity than student assessment revealed in the interaction of student characteristics (mathematics: *t*(884) = 8.32, *p* < .0001, Δ = 0.54, language arts: *t*(785) = 9.82, *p* < .0001, Δ = 0.65).

**Table 3:** Diversity measures based on student assessment and teacher perception for mathematics and language arts.

	N	K	K / K_max_	H	J	Var(H)
*Student Assessment*						
Mathematics	420	59	0.73	3.71	0.91	0.002
Language Arts	446	74	0.91	3.98	0.92	0.002
*Teacher Perception*						
Mathematics	472	44	0.54	3.17	0.84	0.003
Language Arts	459	52	0.64	3.33	0.84	0.003

The number of measured / perceived diversity patterns K of K_max_ = 81 possible diversity patterns. Diversity index H is compared to its theoretical maximum in H / H_max_.

T test results for academic subject area comparison indicated a significant difference in diversity between mathematics and language arts from a student’s point of view (cf. Table 4, *t*(905) = 4.88, *p* < .0001, Δ = 0.27). However, between-subject area comparison on the teachers’ side showed only a small difference between mathematics and language arts teachers that was not significant after accounting for multiple comparisons (*t*(884) = 2.24, *p* < .05, Δ = 0.16).

**Table 4:** Diversity measure comparison between student assessment and teacher perception and between academic subject areas in modified t tests.

	t	df	p
*Student Assessment vs*. *Teacher Perception*			
Mathematics	8.32	884	****
Language Arts	9.82	785	****
*Mathematics vs*. *Language Arts*			
Student Assessment	4.88	905	****
Teacher Perception	2.24	884	*

Significance levels are marked.

* p < .05

** p < .01

*** p < .001

**** p < .0001 (To account for multiple comparisons, only results at p < .01 and smaller are regarded to be significant.)

#### Uncovering types: Frequency analysis of diversity patterns

**Fig 1:**
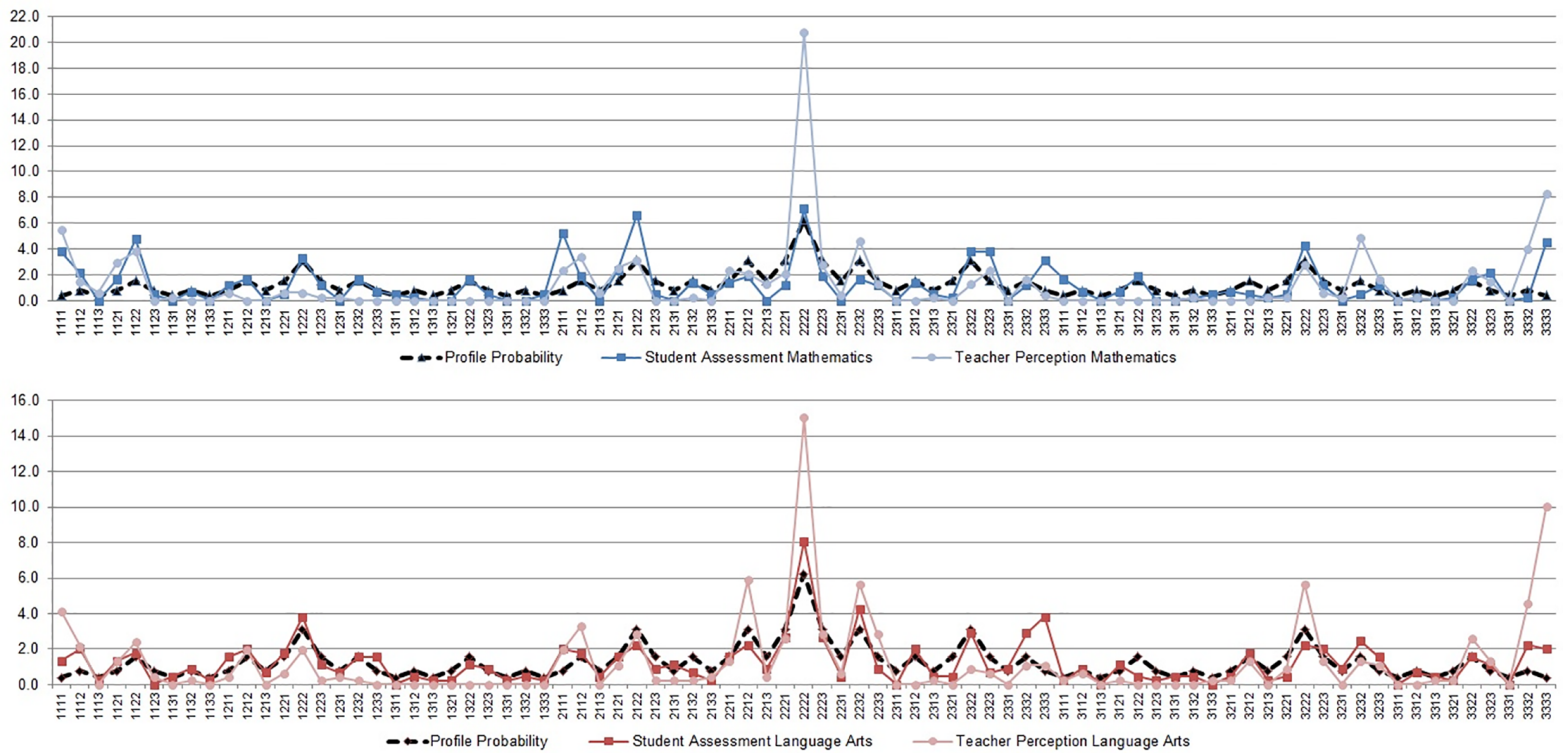
Frequencies of diversity patterns of student characteristics. Observed occurrences of 81 diversity patterns according to student assessment (solid dark, square) and teacher perception (solid light, circle) compared to each other and to theoretical profile probabilities (dashed black, diamond) for mathematics (blue, upper image) and language arts (red, lower image).

Observed frequencies of diversity patterns from students and teacher perspectives are compared to their theoretical probability in Fig 1. More detail is given in Table C in S1 Appendix.

From a student perspective, three diversity patterns where observed significantly more often than expected for mathematics instruction (cf. Table 5): Pattern 3333 (*n*_*o*_ = 19, χ^2^ = 162.70, *p* < .0001), the pattern of overall strong students who had high cognitive ability, high achievement, high interest, and high self-concept; Pattern 1111 (*n*_*o*_ = 16, χ^2^ = 55.00, *p* < .0001), overall weak students; and Pattern 2111 (*n*_*o*_ = 22, χ^2^ = 54.42, *p* < .0001). For language arts, no diversity pattern appeared significantly more often than expected on the students’ side.

**Table 5:** Types of student characteristic patterns in mathematics uncovered by configural frequency analysis.

	Types	n	exp.(n+1)	Q	χ^2^	p
*Student Assessment*						
Mathematics	3 3 3 3	19	1.99	0.04	162.70	****
	1 1 1 1	16	3.38	0.03	55.00	****
	2 1 1 1	22	5.58	0.04	54.42	****
Language Arts	*—no types—*					
*Teacher Perception*						
Mathematics	3 3 3 3	39	2.55	0.07	549.59	****
	1 1 1 1	26	1.52	0.05	427.16	****
	2 2 2 2	98	36.40	0.12	107.63	****
	1 1 2 1	14	3.21	0.02	43.33	***
Language Arts	3 3 3 3	46	3.27	0.08	585.06	****
	1 1 1 1	19	1.21	0.03	292.31	****
	2 2 2 2	69	33.07	0.07	41.24	***

Significance levels of local χ^2^ tests

*** p < .001

**** p < .0001

The teachers’ perspective showed that mathematics teachers perceived four student diversity patterns significantly more frequently than expected: Patterns 3333 and 1111, the overall strong and the overall weak student (*n*_*o*_ = 39, χ^2^ = 549.59, *p* < .0001 and *n*_*o*_ = 26, χ^2^ = 427.16, *p* < .0001); Pattern 2222, students with overall medium student characteristics (*n*_*o*_ = 98, χ^2^ = 107.63, *p* < .0001); and Pattern 1121 (*n*_*o*_ = 14, χ^2^ = 43.33, *p* < .001). In language arts, teachers perceived three student characteristics diversity patterns significantly more frequently than expected: overall strong students, Profile 3333 (*n*_*o*_ = 46, χ^2^ = 585.06, *p* < .0001); overall weak students, Profile 1111 (*n*_*o*_ = 19, χ^2^ = 292.31, *p* < .0001); and overall average students, Profile 2222 (*n*_*o*_ = 69, χ^2^ = 41.24, *p* < .001).

## Discussion

In comparing students’ and teachers’ perspectives on the within-student interaction of different cognitive and motivational-affective characteristics, our study had four major findings: First, students’ reports revealed much diversity in the way characteristics interacted. Second, teachers did not perceive the same extent of diversity in this interplay. Third, detailed finding on student characteristics diversity must be considered academic subject-specific. And fourth, our person-centered methodology allowed a new look onto this diversity.

### Discussion of central findings

First, looking from a student perspective, all three methodological approaches revealed considerable diversity in within-student characteristics. The moderate to low pairwise variable associations agreed with other variable-centered studies finding medium to loose connections between several of the student characteristics [3,7]. In more detail, with the exception of self-concept regarding mathematics, pairwise multi-level random slope regressions showed that student characteristics only seemed to be loosely tied according to student assessment. Especially the weak connection within the cognitive domain, i.e. the relationship between general cognitive ability and prior achievement, was noticeable and seemed to differ from other studies’ findings of a strong predictive power of general cognitive ability on achievement (0.61 ≤ b ≤ 0.77 for languages and mathematics [1]). However, interpretations must consider that within our sample of high-track students, average cognitive ability was above age-group standard. Hence, on a high level, this strong predictive power seemed to abate. This notion should be kept in mind when considering the teachers’ perspective. Overall, however, the agreement of our variable-centered approach with prior research makes our subsequent findings especially interesting: They reveal what low variable connections like these might mean for individual students: Results from both person-centered approaches from a student perspective (the high diversity index and few dominant student patterns) supported the notion of incoherences in the interplay of student characteristics. This was in line with our first hypotheses and with those findings indicating that cognitive and motivational-affective student characteristics do not have to be parallel, cf. [5,6,7]. In more detail, our diversity index results showed that there was a high variability of how cognitive ability, achievement, interest, and self-concept interacted for individual students. Beyond other person-centered studies’ findings that groups of heterogeneous students exist alongside groups of more homogeneous students [5,6], the diversity index illustrated the large extent of diversity when *not* focusing on grouping students. Still, our findings agreed with this line of research in that many students exhibit incoherences in the interplay of their characteristics. One of the unique aspects of our study was considering both, student and teacher perspectives, which other person-centered studies had not yet done, cf. [5,6,7]. Thus, we could directly observe if these incoherences were what teachers noticed about their students’ characteristics interplay (see our second finding below).

Our second finding was that teachers did not perceive the same amount of within-student diversity in the interaction of characteristics as found in student assessment. This was indicated by all three methodological approaches–highlighting different aspects. First, on a variable level, teachers in both academic subject areas perceived moderate to strong connections between general cognitive ability, prior achievement, interest, and self-concept. This was in line with our hypothesis and agreed with other studies’ findings that teachers’ perception of one student characteristic was influenced by their view of another [40,41] and the more general tendency to overgeneralize as described by the halo effect [13,42]. Again, our person-centered methodology gave more detail on these findings: The juxtaposition of the teacher and student perspectives was studied in depth–and by statistical comparisons. Diversity index comparisons found that teachers perceive *significantly* less diversity than student assessment data exhibited. The profound gap between these two perspectives has to be discussed since concepts such as individualized instruction and adaptive teaching build on the idea that teacher and student perceptions of learning characteristics align [8]. Our findings add weight to the conception that judgment of individual differences in the interplay of student characteristics is, indeed, difficult for teachers as research on the complexity of those judgment processes indicates [13]. While our study did not directly assess any of the sources of these biases such as teacher knowledge and beliefs directly, a detailed look into our variable-centered findings gave hints to how ambiguous their role might be in perceiving the interaction of student characteristics. Variable connections within the cognitive domain were strongest for both groups of teachers, which did not correspond to findings from student assessment. Since the strong connection between general cognitive ability and achievement is also established in educational research, cf. [1], we might conjecture that teachers knew about this strong relationship. But without also factoring in knowledge of pseudo-concurrences [4] and neglecting the special case of overall above average levels of general cognitive ability in the highest school track, this knowledge might mislead teachers’ perceptions. Person-centered results, then, show how those biases look across variables for teachers’ look onto their students: Our analyses revealed that teachers predominantly perceived homogeneous types of student characteristics patterns: overall strong and overall weak students as well as an overall average type. Hence, teachers’ internal categorization seemed to view many students as uniform–on different levels. Especially interesting is the predominance of the overall average student (over 20% of students were assigned to this pattern by mathematics teachers) which was not found to be over-frequented in student data for either academic subject area. Its high recurrence in teacher perception seems to give first empirical substantiation to the general observation that in discourses about students “terms like ‘normal,’ ‘typical,’ and ‘average’ are abundant” [14]. Our findings show that this averaging of individual differences seems to not only happen for single characteristics between students but also for interplays of within-student characteristics.

Our third finding supported the idea of academic subject-specificity of student characteristics diversity. While main findings are similar for the two different subject areas, we also uncovered important differences. First, we found overall weaker connections between characteristics, significantly more diversity, and no dominant student pattern types when regarding student assessment in language arts. On a variable level, this was in line with our hypotheses and other studies findings of a closer tie between student characteristics for mathematics compared to language arts [1,21,23]. However, it was remarkable that we were able to show that differences in diversity where statistically highly significant. We conjecture the broader scope of language arts as an academic subject area to play a role [58] where interest or self-concept of students might vary depending on the aspects considered (e.g. analyzing the structure of a poem versus engaging in a collaborative discussion). Another difference was self-concept regarding mathematics, which was the one exception to the weak connections of student characteristics. These moderate connections were not the case for language arts, where they were equally weak as other pairwise associations. Even though academic subject area differences are known [23], such clear between-subject area differences call for more systematic research in comparing academic subjects. Furthermore, the lack of over-frequented diversity patterns in language arts affirms that here, student characteristics diversity is higher in the extremes. An awareness of this fact can be a challenge and a chance for language teachers. Overall, teacher data did not show academic subject area differences as clearly compared to student data. This encourages the understanding that teacher judgment decisions regarding student characteristics diversity is not (as) academic subject-specific. A reason for the differences in subject area-specificity of teacher perception versus student assessment might lie in frame of reference effects. While teachers see many students in one academic subject area, students see themselves in different academic subject areas. This might lead to incoherences in the interplay of student characteristics that teachers do not see.

Finally, reflecting on the methodology of our study, which considered the interplay of student characteristics in a novel way, our findings did not only largely support current research, but uncovered important additional aspects of students’ and teachers’ perspective on the within-student interaction of characteristics. Complementing the analysis of student characteristics interaction on a variable level, e.g. [3], our person-centered approaches uncovered what is behind loose pairwise connections. Configural frequency analyses of students’ characteristics patterns identified which configurations where dominant despite overall low associations (e.g. the overall strong and overall weak students in mathematics). This can help researchers to understand the variable connections better and to identify small subsets of students interesting for further examination, for instance, in form of qualitative investigations of their individual perspectives on specific characteristics and their interplay. This study’s approach also complemented the research on grouping students, e.g. [6], since it uncovers the entire diversity in interacting characteristics. Our methodological expedition to diversity measurement of other disciplines yielded a methodological advance for educational science.

### Limitations and future research

Limitations and ideas for future research can also be discussed along the lines of our four major findings. Firstly, focusing on the students’ side, the agreement of our findings on high student characteristics diversity with other person-centered approaches [5,6] further highlights the significance of studying individual differences–and especially incoherences–within students’ sets of characteristics. Thereby, our study only considers one certain point in time. To comprehend incoherences better, we need to get a better understanding of how they have developed and how this development is connected to student learning. First steps have been taken for specific groups of students: learners with high cognitive abilities and low self-concept showed three different developmental paths over a school year–and for a fourth of students, this incoherence disappeared [59]. In that study, development was connected to student’s internal learning processes. However, it is still unclear if and under which conditions incoherent profiles in general can be temporary phenomena. We need to know whether incoherences are due to developmental delays where certain characteristics are still forming while others are already fully developed. Considering Vygotsky’s theory of proximal development [60], future research ought to identify paths of development for different characteristics configurations guided by teacher scaffolding. Further research that tracks the development of student characteristics over several school years is needed to shed more light onto this issue. Furthermore, it would be enlightening to study students’ perspective on student characteristics diversity in different educational settings. For this study, only students of one academic track (Gymnasium) were considered since cross-track level comparisons might have introduced bias in both, student assessment and teacher perception [61]. However, other educational settings with more heterogeneous placement might uncover even more characteristics diversity. It is remarkable, nevertheless, how much individual difference and diversity is already found within one track of theoretically more homogeneous students.

Secondly, our findings on the teacher perspective must also mention limitations that yield further research. Our study cannot rule out the many sources of bias influencing teacher judgments research mentions [13]. Also, due to our study design where we obtain information on the students’ prior grades through their teachers, we cannot obviate teachers’ judgments being swayed by that information. However, since these were grades given by the teachers of the prior academic year, current teachers might be a little less susceptible to this bias. The study design was chosen this way to avoid the inaccuracy often introduced when students report their own prior grades [62]. Nevertheless, further research might consider assessing students’ prior achievement independently. Furthermore, our study did not assess teachers’ knowledge and beliefs and did not connect judgments to student background like migration or gender. Overall, our findings stand by what other studies on teacher judgments have called for: Further research should address student background diversity and its connection to many possible biases in judgment in teacher education [38].

Regarding teachers’ perceptions, our study also yields many other directions for future research. Considering the predominance of homogeneous types of student characteristics patterns perceived by teachers, it would be interesting to consider contextual factors next as critical research on the halo effect suggests [63]. An interesting step would be to study how teachers interact with those different groups of students–and with the students whose characteristics they perceive more distinctly. Further research is also needed in linking the established approach of teacher perception accuracy to the concept of diversity perception introduced in this paper: Are teachers who perceive less diversity in the patterns of their students’ characteristics also less accurate in their judgment of all single characteristics? Or do they accurately judge prior achievement and generalize this perception to interest or cognitive ability [40,41]? Furthermore, large variations in meta-analysis studies indicate that teachers vary in their ability to accurately assess single student characteristics [11]. Hence, further research must also examine the variance in teachers’ diversity perception and study its conditions. For example, do expert teachers accurately perceive more diversity than novice teachers? Furthermore, studies must regard the consequences of potential differences in teacher diversity perception. Teacher expectations are long known to influence how teachers interact with their students in the classroom [64]. Since diversity indices provide are a way to quantify teacher’s perception of their body of students, further research ought to explore the link between variation in diversity perception and classroom activities. Do teachers who perceive more diversity also engage more students or students more equally in classroom discussions? Do they use a wider range of different activities? Both of which are seen as aspects in adaptive teaching [35]. On the other hand, which in-class situations shape teacher perceptions? One of the most interesting directions in this line of further research is the look for proximal indicators that might trigger teacher judgments, such as student behavior and teacher-student interactions. Research has identified that classroom activities play an important role in teacher judgments [40,65]. First studies have considered this issue using innovative measurement methods, such as eye-tracking [45]. They found that it takes experience for teachers to be able to attend to all students’ input and behavior in class. Correspondingly, research finds, that especially novice teachers struggle when having to attend to complex and dynamic visual stimuli [66]. These findings provide first insights into aspects of the judgment process of teachers. Yet, more research needs to be done.

Thirdly, regarding differences between academic subject areas, further academic subject area comparisons lined with knowledge or beliefs on teacher and student side that are academic subject-specific are necessary to further understand the role this context plays for state and development of student characteristics. Research has found that frame of reference issues can lead to bias in judgment [67]. Future research should identify if this bias plays a role in student characteristics diversity. Regarding the gender-specific connotation of the two respective academic subject areas investigated in our study [15], a possible gender effect in teacher perceptions and student’s self-perceptions seems possible. Hence, further research studying the role of gender in the interplay of student characteristics could provide additional insights.

Finally, from a methodological angle, future research could dig even deeper. Our ability to measure the amount of diversity with regard to a set of characteristics in a student population provides educational researchers with a new methodological tool to examine and account for student characteristics diversity. Foremost, using diversity index comparisons allows for statistically sound comparisons of diversity in different settings, populations, or perspectives. Apart from student diversity indicators that focus on student background information, diversity indices can give other important context information when studying differences between groups of students. Furthermore, with a greater number of participants, configural frequency analyses could uncover anti-types, patterns that occur less frequently than expected and advanced models within configural frequency analysis (e.g. second order or interaction models) can shed further light onto the nature of the relationship among variables, enhancing our understanding of student characteristics interplay even further [68].

### Practical implications

Especially our findings on the teachers’ perspective and their limited percipience of diversity have practical implications for teacher education and professional development. They suggest that addressing the difficulty of teacher judgment as a central theme in teacher education and professional development is crucial. As our study was one of the few regarding teacher judgments that focused on the teacher perception on the within-student interaction of characteristics, the gap found between teacher and student perspectives especially calls for education and professional development to highlight possible incoherences of within-student characteristics. In addition, in-depth discussion on the role of each single cognitive and motivational-affective student characteristics on learning might help teachers recognize and diagnose with more differentiation. Moreover, the role of biases in judgment such as knowledge and beliefs of teachers on student characteristics and their interaction must be substantiated in educational programs. Furthermore, these programs must not only provide current research on the interplay of student characteristics, but also raise awareness for their limitations when applying to individual students as cases and discuss how they apply to teachers’ practice. Finally, teachers must be handed tools that help them focus on the students’ perspective on different student characteristics during instruction. Different modern forms of formative assessment such as clicker systems can, for instance, provide on-the-spot feedback on pre-knowledge but also motivational-affective characteristics regarding instruction [69,70].

### Conclusions

Overall, our study provided a new perspective onto the interplay of different cognitive and motivational-affective student characteristics that are crucial for student learning. In exploring both, the student and the teacher perspective, it found that while there was much diversity in how students’ tested and reported characteristics interact, teachers predominantly saw more homogeneous “overall strong”, “overall weak”, and especially, “overall average” students. This was true for both academic subject areas considered, mathematics and language arts, but detailed findings uncovered important subject area differences as well. The novel methodology showed that educational research must look beyond variable-centered methodology when regarding student characteristics diversity. After all, it is research’s task to provide empirical evidence for the individual differences in students that teachers face in their teaching. Only then can it aid in striving towards individual learning success.

## Supporting information

S1 Appendix (DOCX)

S1 Data Anonymized dataset. (XLSX)
