# Supplementary material for: Comparing teacher and student perspectives on the interplay of cognitive and motivational-affective student characteristics
Source: PLoS One. 2018 Aug 15;13(8):e0200609. doi: 10.1371/journal.pone.0200609 (PMC6093607; doi:10.1371/journal.pone.0200609)
Supplement: S1 Appendix — (DOCX) [file pone.0200609.s001.docx]

# S1 Appendix

## Data analysis

**Pairwise multi-level regressions**

Analyses were performed using the statistical software R [71] and the packages *lme4* [72] and *lmerTest* [73].

For each pair of student characteristics, we compared model fits of the simple regression model

$$Y_{i}=\gamma_{00}+\gamma_{10}X_{i}+r_{i},$$

with $\left( X_{i},Y_{i} \right)$ the pair of student characteristics and $\mathrm{Var}\left( r_{i} \right)=\sigma^{2}$ for student $i$, the multi-level random intercept regression model

$$Y_{\mathrm{ij}}=\beta_{0j}+\beta_{1j}X_{\mathrm{ij}}+r_{ij},$$

$$\beta_{0j}=\gamma_{00}+u_{0j},$$

$$\beta_{1j}=\gamma_{10},$$

with $\left( X_{\mathrm{ij}},Y_{\mathrm{ij}} \right)$ the pair of student characteristics, $\mathrm{Var}\left( r_{\mathrm{ij}} \right)=\sigma^{2}$, and $\mathrm{Var}\left( u_{0j} \right)=\tau_{00}$ for student $i$ in classroom $j$, and the multi-level random slope regression model

$$Y_{\mathrm{ij}}=\beta_{0j}+\beta_{1j}X_{\mathrm{ij}}+r_{\mathrm{ij}},$$

$$\beta_{0j}=\gamma_{00}+u_{0j},$$

$$\beta_{1j}=\gamma_{10}+u_{1j}$$

with $\left( X_{\mathrm{ij}},Y_{\mathrm{ij}} \right)$ the pair of student characteristics, $\mathrm{Var}\left( r_{\mathrm{ij}} \right)=\sigma^{2}$, $\mathrm{Var}\left( u_{0j} \right)=\tau_{00}$, $\mathrm{Var}\left( u_{1j} \right)=\tau_{11}$, and $\mathrm{Cov}\left( u_{1j},u_{0j} \right)=\tau_{10}$ for student $i$ in classroom $j$, to find the most appropriate model describing this relationship while considering the multi-level structure of our data wherever appropriate. Table A gives an overview over the comparative model fit and indicates which model was chosen for each pair. Full regression results of the chosen models are given in Table B. For simple regressions, we report the fixed effects with standard error and significance of corresponding t tests as well as the model’s results of the F test. For multi-level random intercept and random slope models, we additionally provide variances of group and residual. To account for multiple comparisons, significance levels were adjusted using a Bonferroni correction [74] of p’ = p / m, with m being the number of tests run. Hence, for the 48 pairwise multi-level regressions, since p’ = 0.05 / 48 ≈ 0.001, results at p < .001 and smaller are regarded to be significant.

**Insert Table A here.**

**Insert Table B here.**

Regarding the measurement level of each $\left( X_{\mathrm{ij}},Y_{\mathrm{ij}} \right)$ pair of student characteristics, researchers have discussed the appropriateness of using analyses like regression analysis on ordinal integer scale variables and many authors conclude that the disadvantages of only obtaining approximations (since assumptions like the normality of residuals are somewhat violated) can be outweighed by the benefits of gaining results that are familiar and easy to interpret while often not being too inexact [75,76].

**Diversity index H and modified t tests**

The Shannon-Wiener diversity is founded on the Shannon entropy, which was initially developed by C. E. Shannon in 1948 [77]. While there are other measures of diversity [e.g. 78; an overview can be found in 54], Shannon’s formula holds a central position when compared to other diversity measures [79]. The Shannon-Wiener diversity index *H,* known to measure diversity, for instance, in biology, ecology, and forestry, was calculated as $H = - \sum_{k=1}^{K} p_{k} ln(p_{k})$, where $p_{k}$ is the observed proportion of individuals with diversity pattern *k* and *K* is the number of total patterns present according to student assessment and teacher perceptions in either academic subject area. The diversity index increases when there are more different species and when they appear more equally distributed. Connected to the diversity index is a measure of evenness, $J=H/{H_{max}}$, relating *H* to its maximum $H_{max}=ln\left( K \right)$. As our application also refers to diversity measurement, we refer to this name instead of the more general entropy. Diversity indices can be compared across samples using Hutcheson’s student's t test [80,81] with the variance

$$\mathrm{var}\left( H \right)=\frac{\sum_{k=1}^{K} p_{k}\left[ ln(p_{k}) \right]^{2}-\left[ \sum_{k=1}^{K} p_{k} ln(p_{k}) \right]^{2}}{N}+\frac{K-1}{2N^{2}}$$

calculated for both diversity indices H1 and H2 to be compared, the test statistic

$$t=\frac{H_{1}-H_{2}}{\sqrt{\mathrm{var}\left( H_{1} \right)+var\left( H_{2} \right)}},$$

degrees of freedom

$$df=\frac{\left[ \mathrm{var}\left( H_{1} \right)+var\left( H_{2} \right) \right]^{2}}{\frac{\left[ \mathrm{var}\left( H_{1} \right) \right]^{2}}{N_{1}}+\frac{\left[ \mathrm{var}\left( H_{2} \right) \right]^{2}}{N_{2}}}$$

and the effect size

$$\Delta=\left| H_{1}-H_{2} \right|$$

of this comparison. Results of t tests can be found in Table 4 (of the above research paper). To account for multiple comparisons via Bonferroni correction [74], since p’ = 0.05 / 4 ≈ 0.01, results at p < .01 and smaller are regarded to be significant. All calculations were performed in MS Excel and, where reliable implementation in R was available, results were verified there using the package *vegan* [82].

**Frequencies and ranking of diversity patterns**

Configural frequency analyzes (CFA) were performed in R's *cfa* package [83]. This study used the original CFA base model, also known as first order CFA or model of variable independence. This does not mean that we assume our four student characteristics to be independent of each other, rather that the expected frequencies are defined according to this null hypothesis or base model (i.e. the assumption of independence) and types emerge due to any associations among those variables [27,68]. Thus, it allows us to study these connections among the student characteristics from a person-centered view. Types in configural frequency analysis can be identified employing local χ2 tests relating observed frequencies (n_o_) to expected frequencies (n_e_) [55]:

$$\chi^{2}=\frac{\left( n_{o}-n_{e} \right)^{2}}{n_{e}}$$

with df=1. Observed frequencies of diversity patterns and their respective ranking order in configural frequency results based on local χ^2^ tests are given in Table C.

**Insert Table C here.**

# References

1. R Core Team (2014). R: A language and environment for statistical computing. R Foundation for Statistical Computing, Vienna, Austria. URL http://www.R-project.org/.
2. Bates D., Maechler M., Bolker B. & Walker S. (2014). *lme4: Linear mixed-effects models using Eigen and S4.* R package version 1.1-7, URL: http://CRAN.R-project.org/package=lme4.
3. Kuznetsova, A., Brockhoff, P. B., & Christensen, R. H. (2015). *lmerTest: Tests in Linear Mixed Effects Models. R package version 2.0-25* (http://CRAN.R-project.org/package=lmerTest).
4. Shaffer, J. P. (1995) Multiple Hypothesis Testing. *Annual Review of Psychology, 46*, 561-584.
5. Kim, J.-O. (1975). Multivariate Analysis of Ordinal Variables. *American Journal of Sociology, 81*(2), 261-298.
6. Labovitz, S. (1967). Some observations on measurement and statistics. *Social Forces, 46*, 151-160.
7. Shannon, C. E. (1948). A Mathematical Theory of Communication. *The Bell System Technical Journal, 3*(27), 379-423.
8. Simpson, E. H. (1949). Measurement of Diversity. *Nature, 163,* 688.
9. Laxton, R. R. (1978). The Measure of Diversity. *Journal of Theoretical Biology, 70,* 51–67.
10. Zar, J. H. (2013). *Biostatistical Analysis* (5th ed.)*.* Essex: Pearson Education.
11. Hutcheson, K. (1970). A test for comparing diversity based on the Shannon formula. *Journal of Theoretical Biology,* *29,* 151–154.
12. Oksanen, J. F., Blanchet, G., Kindt, R., Legendre, P., Minchin, P. R., O'Hara, R. B. et al. (2015). *vegan: Community Ecology Package.* R package version 2.3-0. http://CRAN.R-project.org/package=vegan
13. Funke, S., Mair, P., von Eye, A., & Harloff, J. (2013). *cfa: Analysis of Configuration Frequencies (CFA). R package version 0.9-3* (http://CRAN.R-project.org/package=cfa).

**Table A. Model fit of simple, multi-level random intercept, or multi-level random slope regression model for pairs of student characteristics.**

|  |  |  | Simple regression model  (df = 3) | |  | Multi-level random intercept model  (df = 4) | | |  | Multi-level  random slope model  (df = 6) | | |
| --- | --- | --- | --- | --- | --- | --- | --- | --- | --- | --- | --- | --- |
|  |  |  | χ^2^ | × |  | χ^2^ | p | × |  | χ^2^ | p | × |
| *Student Assessment:*  *Mathematics* |  |  |  |  |  |  |  |  |  |  |  |  |
| G. Cognitive Ability | ACH |  | 1164.87 | × |  | 1162.27 | .107 |  |  | 1162.26 | .992 |  |
|  | INT |  | 1268.66 | × |  | 1267.07 | .208 |  |  | 1265.41 | .435 |  |
|  | SC |  | 1265.33 |  |  | 1262.32 | .083 | × |  | 1262.11 | .902 |  |
| Achievement | GCA |  | 1170.03 | × |  | 1167.38 | .104 |  |  | 1167.21 | .919 |  |
|  | INT |  | 1188.69 | × |  | 1188.10 | .440 |  |  | 1188.04 | .975 |  |
|  | SC |  | 1105.66 | × |  | 1105.66 | 1.00 |  |  | 1105.66 | 1.00 |  |
| Interest | GCA |  | 1272.27 |  |  | 1257.12 | .000 | × |  | 1253.87 | .197 |  |
|  | ACH |  | 1182.24 |  |  | 1167.82 | .000 | × |  | 1167.17 | .723 |  |
|  | SC |  | 1166.05 |  |  | 1161.01 | .025 | × |  | 1158.37 | .267 |  |
| Self-concept | GCA |  | 1265.67 |  |  | 1253.29 | .000 | × |  | 1253.25 | .979 |  |
|  | ACH |  | 1102.33 |  |  | 1095.91 | .011 | × |  | 1093.37 | .281 |  |
|  | INT |  | 1166.12 | × |  | 1164.31 | .178 |  |  | 1162.69 | .446 |  |
| *Student Assessment:*  *Language Arts* |  |  |  |  |  |  |  |  |  |  |  |  |
| G. Cognitive Ability | ACH |  | 1248.93 | × |  | 1247.46 | .226 |  |  | 1246.17 | .523 |  |
|  | INT |  | 1280.99 | × |  | 1279.80 | .274 |  |  | 1276.67 | .209 |  |
|  | SC |  | 1283.39 | × |  | 1281.74 | .199 |  |  | 1280.84 | .637 |  |
| Achievement | GCA |  | 1244.14 |  |  | 1239.23 | .027 | × |  | 1238.86 | .835 |  |
|  | INT |  | 1269.06 |  |  | 1261.22 | .005 | × |  | 1259.63 | .452 |  |
|  | SC |  | 1250.81 |  |  | 1243.92 | .009 | × |  | 1242.78 | .568 |  |
| Interest | GCA |  | 1270.06 |  |  | 1269.03 | .310 |  |  | 1262.68 | .042 | × |
|  | ACH |  | 1273.98 |  |  | 1268.99 | .026 | × |  | 1268.27 | .696 |  |
|  | SC |  | 1315.12 |  |  | 1310.64 | .034 | × |  | 1310.04 | .739 |  |
| Self-concept | GCA |  | 1288.61 | × |  | 1285.66 | .086 |  |  | 1285.32 | .841 |  |
|  | ACH |  | 1255.98 |  |  | 1250.05 | .015 | × |  | 1249.68 | .831 |  |
|  | INT |  | 1315.43 |  |  | 1309.47 | .015 |  |  | 1303.27 | .045 | × |
| *Teacher Perception:*  *Mathematics* |  |  |  |  |  |  |  |  |  |  |  |  |
| G. Cognitive Ability | ACH |  | 1054.62 |  |  | 1037.23 | .000 |  |  | 1031.46 | .056 | × |
|  | INT |  | 1164.73 |  |  | 1156.40 | .004 | × |  | 1153.56 | .242 |  |
|  | SC |  | 1210.38 |  |  | 1199.70 | .001 | × |  | 1198.68 | .600 |  |
| Achievement | GCA |  | 1058.68 |  |  | 1020.00 | .000 | × |  | 1018.46 | .463 |  |
|  | INT |  | 1197.82 |  |  | 1166.72 | .000 | × |  | 1163.48 | .197 |  |
|  | SC |  | 1190.47 |  |  | 1178.36 | .001 | × |  | 1185.32 | 1 |  |
| Interest | GCA |  | 1170.62 |  |  | 1158.39 | .000 | × |  | 1156.85 | .464 |  |
|  | ACH |  | 1197.70 |  |  | 1185.13 | .000 | × |  | 1185.11 | .991 |  |
|  | SC |  | 1274.75 |  |  | 1266.33 | .004 | × |  | 1263.46 | .237 |  |
| Self-concept | GCA |  | 1212.03 |  |  | 1167.29 | .000 | × |  | 1162.68 | .100 |  |
|  | ACH |  | 1188.58 |  |  | 1164.04 | .000 |  |  | 1156.38 | .022 | × |
|  | INT |  | 1272.23 |  |  | 1233.95 | .000 |  |  | 1210.55 | .000 | × |
| *Teacher Perception:*  *Language Arts* |  |  |  |  |  |  |  |  |  |  |  |  |
| G. Cognitive Ability | ACH |  | 1080.22 |  |  | 1076.18 | .044 |  |  | 1067.91 | .016 | × |
|  | INT |  | 1207.65 | × |  | 1206.95 | .402 |  |  | 1205.36 | .452 |  |
|  | SC |  | 1215.59 |  |  | 1209.83 | .016 |  |  | 1198.83 | .004 | × |
| Achievement | GCA |  | 1082.88 | × |  | 1081.18 | .193 |  |  | 1077.46 | .156 |  |
|  | INT |  | 1198.89 |  |  | 1197.78 | .293 |  |  | 1189.53 | .016 | × |
|  | SC |  | 1172.59 | × |  | 1170.09 | .114 |  |  | 1168.31 | .411 |  |
| Interest | GCA |  | 1210.50 |  |  | 1194.96 | .000 | × |  | 1194.55 | .814 |  |
|  | ACH |  | 1198.72 |  |  | 1176.66 | .000 |  |  | 1167.79 | .012 | × |
|  | SC |  | 1194.64 |  |  | 1182.44 | .000 | × |  | 1181.65 | .674 |  |
| Self-concept | GCA |  | 1215.92 |  |  | 1169.45 | .000 |  |  | 1162.17 | .026 | × |
|  | ACH |  | 1170.27 |  |  | 1125.49 | .000 | × |  | 1123.24 | .324 |  |
|  | INT |  | 1192.12 |  |  | 1160.69 | .000 |  |  | 1153.44 | .027 | × |

Chosen model is indicated. Cognitive domain: GCA: general cognitive ability, ACH: achievement; motivational-affective domain: INT: interest, SC: self-concept. p: significance of χ2-difference for this model compared to the next simpler one. × : The model was chosen for this pair of characteristics.

**Table B. Full model results of simple, multi-level random intercept, or multi-level random slope regression models.**

|  |  |  | Fixed effects | |  | Random effects | |  |  |  |
| --- | --- | --- | --- | --- | --- | --- | --- | --- | --- | --- |
|  | Predictor |  | $\gamma_{00}$ (SE) | $\gamma_{10}$  (SE) |  | $\tau_{00}$ | $\sigma^{2}$ |  | F(df_1_,df_2_) | p |
| *Student Assessment: Mathematics* |  |  |  |  |  |  |  |  |  |  |
| GCA | ACH |  | -0.02 (0.05) | 0.23*** (0.05) |  |  |  |  | F(1,418)=24.02 | **** |
|  | INT |  | 0.00 (0.05) | 0.21*** (0.05) |  |  |  |  | F(1,453)=22.06 | **** |
|  | SC |  | 0.00  (0.06) | 0.24***  (0.05) |  | 0.03 | 0.92 |  | F(1,450)=26.74 | **** |
| ACH | GCA |  | 0.02 (0.05) | 0.23*** (0.05) |  |  |  |  | F(1,418)=24.02 | **** |
|  | INT |  | 0.01  (0.05) | 0.34***  (0.05) |  |  |  |  | F(1,435)=55.05 | **** |
|  | SC |  | 0.01  (0.04) | 0.52***  (0.04) |  |  |  |  | F(1,435)=158.41 | **** |
| INT | GCA |  | -0.01 (0.08) | 0.21*** (0.05) |  | 0.07 | 0.89 |  | F(1,451)=21.00 | **** |
|  | ACH |  | -0.01  (0.07) | 0.33***  (0.04) |  | 0.07 | 0.81 |  | F(1,431)=55.59 | **** |
|  | SC |  | -0.00  (0.05) | 0.54***  (0.04) |  | 0.03 | 0.67 |  | F(1,471)=195.32 | **** |
| SC | GCA |  | -0.01  (0.07) | 0.23***  (0.05) |  | 0.06 | 0.88 |  | F(1,452)=27.20 | **** |
|  | ACH |  | -0.00  (0.06) | 0.50***  (0.04) |  | 0.03 | 0.70 |  | F(1,434)=155.11 | **** |
|  | INT |  | 0.00  (0.04) | 0.55***  (0.04) |  |  |  |  | F(1,470)=207.2 | **** |
| *Student Assessment: Language Arts* |  |  |  |  |  |  |  |  |  |  |
| GCA | ACH |  | -0.02 (0.05) | 0.18*** (0.05) |  |  |  |  | F(1,444)=14.97 | *** |
|  | INT |  | -0.00  (0.05) | 0.13** (0.05) |  |  |  |  | F(1,453)=9.36 | ** |
|  | SC |  | 0.00 (0.05) | 0.12** (0.05) |  |  |  |  | F(1,453)=6.92 | ** |
| ACH | GCA |  | 0.06 (0.06) | 0.18*** (0.05) |  | 0.04 | 0.91 |  | F(1,445)=14.93 | *** |
|  | INT |  | 0.04 (0.07) | 0.26*** (0.04) |  | 0.05 | 0.88 |  | F(1,460)=34.53 | **** |
|  | SC |  | 0.03 (0.06) | 0.32*** (0.05) |  | 0.04 | 0.85 |  | F(1,460)=53.32 | **** |
| INT | GCA |  | 0.03 (0.05) | 0.15* (0.06) |  | 0.01 | 0.90 |  | F(1,455)=5.40 | * |
|  | ACH |  | -0.01 (0.06) | 0.26*** (0.05) |  | 0.04 | 0.90 |  | F(1,460)=34.13 | **** |
|  | SC |  | 0.00 (0.06) | 0.19*** (0.05) |  | 0.04 | 0.93 |  | F(1,469)=16.66 | *** |
| SC | GCA |  | 0.00 (0.05) | 0.12** (0.05) |  |  |  |  | F(1,453)=6.92 | ** |
|  | ACH |  | 0.00 (0.06) | 0.32*** (0.04) |  | 0.04 | 0.86 |  | F(1,460)=53.16 | **** |
|  | INT |  | -0.00 (0.06) | 0.18** (0.05) |  | 0.04 | 0.91 |  | F(1,469)=11.12 | ** |
| *Teacher Perception: Mathematics* | |  |  |  |  |  |  |  |  |  |
| GCA | ACH |  | -0.01  (0.06) | 0.71***  (0.05) |  | 0.04 | 0.48 |  | F(1,475)=213.44 | **** |
|  | INT |  | -0.01  (0.06) | 0.56***  (0.04) |  | 0.04 | 0.64 |  | F(1,475)=224.24 | **** |
|  | SC |  | -0.01  (0.06) | 0.51***  (0.04) |  | 0.04 | 0.72 |  | F(1,456)=156.1 | **** |
| ACH | GCA |  | 0.02 (0.07) | 0.68***  (0.03) |  | 0.07 | 0.47 |  | F(1,469)=429.84 | **** |
|  | INT |  | 0.01  (0.07) | 0.56***  (0.04) |  | 0.08 | 0.60 |  | F(1,483)=233.15 | **** |
|  | SC |  | 0.01  (0.06) | 0.54***  (0.04) |  | 0.04 | 0.66 |  | F(1,465)=187.28 | **** |
| INT | GCA |  | 0.03  (0.06) | 0.57***  (0.04) |  | 0.05 | 0.64 |  | F(1,474)=224.66 | **** |
|  | ACH |  | 0.00  (0.06) | 0.58***  (0.04) |  | 0.04 | 0.64 |  | F(1,484)=231.68 | **** |
|  | SC |  | 0.01  (0.06) | 0.41***  (0.04) |  | 0.04 | 0.80 |  | F(1,456)=92.85 | **** |
| SC | GCA |  | 0.01  (0.08) | 0.48***  (0.04) |  | 0.11 | 0.65 |  | F(1,465)=157.85 | **** |
|  | ACH |  | -0.01  (0.06) | 0.53***  (0.06) |  | 0.05 | 0.61 |  | F(1,479)=91.14 | **** |
|  | INT |  | 0.02  (0.09) | 0.39***  (0.07) |  | 0.12 | 0.66 |  | F(1,479)=33.25 | **** |
| *Teacher Perception:*  *Language Arts* | |  |  |  |  |  |  |  |  |  |
| GCA | ACH |  | -0.01  (0.05) | 0.62***  (0.05) |  | 0.03 | 0.58 |  | F(1,459)=185.98 | **** |
|  | INT |  | 0.01  (0.04) | 0.43***  (0.04) |  |  |  |  | F(1,457)=103.52 | **** |
|  | SC |  | -0.02  (0.06) | 0.43***  (0.06) |  | 0.04 | 0.76 |  | F(1,459)=53.80 | **** |
| ACH | GCA |  | 0.00  (0.04) | 0.62***  (0.04) |  |  |  |  | F(1,457)=282.88 | **** |
|  | INT |  | 0.01  (0.05) | 0.57***  (0.05) |  | 0.01 | 0.66 |  | F(1,486)=136.87 | **** |
|  | SC |  | 0.00  (0.04) | 0.50***  (0.04) |  |  |  |  | F(1,457)=151.52 | **** |
| INT | GCA |  | -0.02  (0.07) | 0.42***  (0.04) |  | 0.06 | 0.76 |  | F(1,454)=103.05 | **** |
|  | ACH |  | 0.01  (0.07) | 0.55***  (0.04) |  | 0.06 | 0.61 |  | F(1,486)=156.29 | **** |
|  | SC |  | -0.02  (0.06) | 0.46***  (0.04) |  | 0.05 | 0.74 |  | F(1,447)=118.85 | **** |
| SC | GCA |  | 0.01  (0.09) | 0.40***  (0.06) |  | 0.13 | 0.67 |  | F(1,459)=52.31 | **** |
|  | ACH |  | 0.00  (0.09) | 0.48***  (0.04) |  | 0.12 | 0.63 |  | F(1,447)=163.12 | **** |
|  | INT |  | -0.00  (0.08) | 0.44***  (0.06) |  | 0.11 | 0.66 |  | F(1,459)=60.83 | **** |

* p < .05, ** p < .01, *** p < .001, **** p < .0001 (To account for multiple comparisons, only results at p < .001 and smaller are regarded to be significant.)

**Table C. Observed frequencies of diversity patterns (n_o_) and their respective ranking based on local χ^2^ tests for student assessment and teacher perception in mathematics and language arts.**

|  |  | Student Assessment | | | |  | Teacher Perception | | | |
| --- | --- | --- | --- | --- | --- | --- | --- | --- | --- | --- |
| Profile | Theoretical Profile Probability | Mathematics | | Language Arts | |  | Mathematics | | Language Arts | |
|  | (N=503) | (N=420) | | (N=446) | |  | (N=472) | | (N=459) | |
|  |  | n_o_ | χ^2^-Rank | n_o_ | χ^2^-Rank |  | n_o_ | χ^2^-Rank | n_o_ | χ^2^-Rank |
| 1111 | 1.96 | 16 | 2 | 6 | 4 |  | 26 | 2 | 19 | 2 |
| 1112 | 3.93 | 9 | 37 | 9 | 5 |  | 7 | 18 | 10 | 5 |
| 1113 | 1.96 | 0 | 27 | 1 | 71 |  | 3 | 26 | 0 | 67 |
| 1121 | 3.93 | 7 | 62 | 6 | 10 |  | 14 | 4 | 6 | 7 |
| 1122 | 7.86 | 20 | 13 | 8 | 58 |  | 18 | 6 | 11 | 12 |
| 1123 | 3.93 | 2 | 23 | 0 | 14 |  | 0 | 46 | 2 | 78 |
| 1131 | 1.96 | 0 | 40 | 2 | 75 |  | 1 | 81 | 0 | 70 |
| 1132 | 3.93 | 3 | 55 | 4 | 63 |  | 0 | 32 | 1 | 42 |
| 1133 | 1.96 | 0 | 35 | 1 | 54 |  | 0 | 67 | 0 | 63 |
| 1211 | 3.93 | 5 | 21 | 7 | 11 |  | 3 | 62 | 2 | 74 |
| 1212 | 7.86 | 7 | 72 | 9 | 72 |  | 0 | 21 | 9 | 51 |
| 1213 | 3.93 | 0 | 28 | 3 | 67 |  | 0 | 55 | 0 | 35 |
| 1221 | 7.86 | 2 | 34 | 8 | 45 |  | 3 | 70 | 3 | 77 |
| 1222 | 15.72 | 14 | 65 | 17 | 44 |  | 3 | 15 | 9 | 54 |
| 1223 | 7.86 | 5 | 77 | 5 | 52 |  | 1 | 36 | 1 | 28 |
| 1231 | 3.93 | 0 | 42 | 3 | 50 |  | 1 | 68 | 2 | 80 |
| 1232 | 7.86 | 7 | 38 | 7 | 30 |  | 0 | 16 | 1 | 13 |
| 1233 | 3.93 | 3 | 60 | 7 | 34 |  | 0 | 44 | 0 | 29 |
| 1311 | 1.96 | 2 | 76 | 0 | 36 |  | 0 | 77 | 0 | 75 |
| 1312 | 3.93 | 1 | 25 | 2 | 32 |  | 0 | 49 | 0 | 34 |
| 1313 | 1.96 | 0 | 39 | 1 | 60 |  | 0 | 76 | 0 | 65 |
| 1321 | 3.93 | 0 | 17 | 1 | 31 |  | 0 | 56 | 0 | 58 |
| 1322 | 7.86 | 7 | 66 | 5 | 38 |  | 0 | 20 | 0 | 17 |
| 1323 | 3.93 | 2 | 45 | 4 | 65 |  | 0 | 53 | 0 | 43 |
| 1331 | 1.96 | 0 | 57 | 1 | 49 |  | 0 | 74 | 0 | 69 |
| 1332 | 3.93 | 0 | 20 | 2 | 19 |  | 0 | 38 | 0 | 27 |
| 1333 | 1.96 | 2 | 71 | 1 | 46 |  | 0 | 73 | 0 | 61 |
| 2111 | 3.93 | 22 | 3 | 9 | 3 |  | 11 | 7 | 9 | 6 |
| 2112 | 7.86 | 8 | 54 | 8 | 64 |  | 16 | 19 | 15 | 14 |
| 2113 | 3.93 | 0 | 15 | 2 | 56 |  | 3 | 80 | 0 | 31 |
| 2121 | 7.86 | 10 | 79 | 7 | 42 |  | 12 | 42 | 5 | 72 |
| 2122 | 15.72 | 28 | 24 | 10 | 53 |  | 15 | 52 | 13 | 73 |
| 2123 | 7.86 | 2 | 8 | 4 | 51 |  | 0 | 11 | 1 | 24 |
| 2131 | 3.93 | 0 | 18 | 5 | 48 |  | 0 | 27 | 1 | 50 |
| 2132 | 7.86 | 6 | 53 | 3 | 9 |  | 1 | 10 | 1 | 11 |
| 2133 | 3.93 | 2 | 51 | 1 | 20 |  | 0 | 24 | 2 | 52 |
| 2211 | 7.86 | 6 | 56 | 7 | 70 |  | 11 | 30 | 6 | 79 |
| 2212 | 15.72 | 8 | 61 | 10 | 24 |  | 10 | 39 | 27 | 21 |
| 2213 | 7.86 | 0 | 16 | 4 | 33 |  | 6 | 79 | 2 | 20 |
| 2221 | 15.72 | 5 | 33 | 12 | 66 |  | 10 | 57 | 12 | 64 |
| 2222 | 31.44 | 30 | 10 | 36 | 6 |  | 98 | 3 | 69 | 3 |
| 2223 | 15.72 | 8 | 70 | 12 | 76 |  | 13 | 75 | 13 | 76 |
| 2231 | 7.86 | 0 | 19 | 2 | 8 |  | 2 | 23 | 3 | 33 |
| 2232 | 15.72 | 7 | 74 | 19 | 73 |  | 22 | 78 | 26 | 60 |
| 2233 | 7.86 | 5 | 68 | 4 | 15 |  | 6 | 63 | 13 | 40 |
| 2311 | 3.93 | 0 | 22 | 0 | 12 |  | 0 | 45 | 0 | 36 |
| 2312 | 7.86 | 6 | 69 | 9 | 61 |  | 0 | 14 | 0 | 9 |
| 2313 | 3.93 | 2 | 58 | 2 | 47 |  | 1 | 59 | 1 | 44 |
| 2321 | 7.86 | 1 | 14 | 2 | 17 |  | 0 | 17 | 0 | 22 |
| 2322 | 15.72 | 16 | 80 | 13 | 77 |  | 6 | 13 | 4 | 10 |
| 2323 | 7.86 | 16 | 5 | 3 | 23 |  | 11 | 41 | 3 | 38 |
| 2331 | 3.93 | 0 | 29 | 4 | 79 |  | 0 | 35 | 0 | 32 |
| 2332 | 7.86 | 5 | 73 | 13 | 35 |  | 8 | 72 | 5 | 30 |
| 2333 | 3.93 | 13 | 4 | 17 | 1 |  | 2 | 64 | 5 | 66 |
| 3111 | 1.96 | 7 | 6 | 1 | 78 |  | 0 | 65 | 1 | 81 |
| 3112 | 3.93 | 3 | 43 | 4 | 69 |  | 0 | 25 | 3 | 56 |
| 3113 | 1.96 | 0 | 30 | 0 | 41 |  | 0 | 61 | 0 | 45 |
| 3121 | 3.93 | 3 | 59 | 5 | 16 |  | 0 | 34 | 1 | 57 |
| 3122 | 7.86 | 8 | 48 | 2 | 13 |  | 0 | 9 | 0 | 8 |
| 3123 | 3.93 | 0 | 12 | 1 | 40 |  | 0 | 31 | 0 | 25 |
| 3131 | 1.96 | 0 | 47 | 2 | 68 |  | 0 | 54 | 0 | 47 |
| 3132 | 3.93 | 1 | 26 | 2 | 28 |  | 1 | 28 | 0 | 15 |
| 3133 | 1.96 | 2 | 78 | 0 | 29 |  | 0 | 51 | 1 | 62 |
| 3211 | 3.93 | 3 | 64 | 2 | 55 |  | 0 | 43 | 1 | 37 |
| 3212 | 7.86 | 2 | 32 | 8 | 74 |  | 0 | 12 | 6 | 26 |
| 3213 | 3.93 | 1 | 63 | 1 | 25 |  | 1 | 58 | 0 | 19 |
| 3221 | 7.86 | 2 | 41 | 2 | 18 |  | 1 | 22 | 4 | 48 |
| 3222 | 15.72 | 18 | 9 | 10 | 43 |  | 13 | 48 | 26 | 41 |
| 3223 | 7.86 | 5 | 81 | 9 | 22 |  | 3 | 37 | 6 | 49 |
| 3231 | 3.93 | 0 | 50 | 4 | 81 |  | 1 | 47 | 0 | 23 |
| 3232 | 7.86 | 2 | 49 | 11 | 57 |  | 23 | 8 | 6 | 16 |
| 3233 | 3.93 | 5 | 11 | 7 | 21 |  | 8 | 33 | 5 | 71 |
| 3311 | 1.96 | 0 | 52 | 0 | 39 |  | 0 | 71 | 0 | 59 |
| 3312 | 3.93 | 1 | 31 | 3 | 59 |  | 1 | 50 | 0 | 18 |
| 3313 | 1.96 | 0 | 46 | 2 | 62 |  | 0 | 69 | 1 | 68 |
| 3321 | 3.93 | 1 | 36 | 1 | 37 |  | 0 | 40 | 1 | 55 |
| 3322 | 7.86 | 7 | 75 | 7 | 80 |  | 11 | 66 | 12 | 53 |
| 3323 | 3.93 | 9 | 7 | 5 | 27 |  | 7 | 29 | 6 | 39 |
| 3331 | 1.96 | 0 | 67 | 0 | 26 |  | 0 | 60 | 0 | 46 |
| 3332 | 3.93 | 1 | 44 | 10 | 7 |  | 19 | 5 | 21 | 4 |
| 3333 | 1.96 | 19 | 1 | 9 | 2 |  | 39 | 1 | 46 | 1 |

χ^2^-Rank is the rank of this diversity pattern based on a comparison of expected and observed frequency considering local χ^2^ tests.
